# Prediction of Quantum Anomalous Hall Effect in MBi and MSb (M:Ti, Zr, and Hf) Honeycombs

**DOI:** 10.1186/s11671-017-2424-y

**Published:** 2018-02-07

**Authors:** Zhi-Quan Huang, Wei-Chih Chen, Gennevieve M. Macam, Christian P. Crisostomo, Shin-Ming Huang, Rong-Bin Chen, Marvin A. Albao, Der-Jun Jang, Hsin Lin, Feng-Chuan Chuang

**Affiliations:** 1 Department of Physics, National Sun Yat-Sen University, Kaohsiung, 804 Taiwan; 2 Multidisciplinary and Data Science Research Center, National Sun Yat-Sen University, Kaohsiung, 804 Taiwan; 3 Center of General Studies, National Kaohsiung Marine University, Kaohsiung, 811 Taiwan; 4 Institute of Mathematical Sciences and Physics, University of The Philippines Los Baños College, Laguna, 811 Philippines; 5 Institute of Physics, Academia Sinica, Taipei, 11529 Taiwan; 6 Centre for Advanced 2D Materials and Graphene Research Centre, National University of Singapore, 6 Science Drive 2, Singapore, 117546 Singapore; 7 Department of Physics, National University of Singapore, 2 Science Drive 3, Singapore, 117542 Singapore

**Keywords:** Quantum anomalous Hall effect, Topological phase transition, TM-Bi honeycomb, Electronic structures, First-principles calculations

## Abstract

The abounding possibilities of discovering novel materials has driven enhanced research effort in the field of materials physics. Only recently, the quantum anomalous hall effect (QAHE) was realized in magnetic topological insulators (TIs) albeit existing at extremely low temperatures. Here, we predict that MPn (M =Ti, Zr, and Hf; Pn =Sb and Bi) honeycombs are capable of possessing QAH insulating phases based on first-principles electronic structure calculations. We found that HfBi, HfSb, TiBi, and TiSb honeycomb systems possess QAHE with the largest band gap of 15 meV under the effect of tensile strain. In low-buckled HfBi honeycomb, we demonstrated the change of Chern number with increasing lattice constant. The band crossings occurred at low symmetry points. We also found that by varying the buckling distance we can induce a phase transition such that the band crossing between two Hf d-orbitals occurs along high-symmetry point K2. Moreover, edge states are demonstrated in buckled HfBi zigzag nanoribbons. This study contributes additional novel materials to the current pool of predicted QAH insulators which have promising applications in spintronics.

## Background

Rigorous research efforts have been continuously focused towards the exploration of novel 2D materials such as quantum spin Hall (QSH) insulators. These novel materials, also known as two-dimensional topological insulators (2D TIs) exhibit a unique property wherein the edges possess spin-polarized gapless states despite the bulk system being an insulator [1]. QSH insulators show dissipationless spin/charge transport which is highly important in spintronic device applications [2]. Recently, it has been discovered that the breaking of time-reversal symmetry (TRS) in QSH insulators lead to a quantum anomalous Hall effect (QAHE) system in which helical edge states are converted into chiral edge states [3]. The dissipationless charge transport without the need for an external magnetic field provides promising applications in low energy consumption spintronics [4, 5] and has encouraged the search for more QAHE systems [6, 7]. Predicted by Haldane in 1988, QAHE was only experimentally achieved in 2013 by magnetically doping thin films of topological insulators [8]. Theoretical studies have suggested that the quantum anomalous Hall (QAH) phase can be achieved by breaking the TRS of a TI by introducing ferromagnetism and inducing a band inversion transition by strong spin-orbit coupling (SOC) effects [9, 10]. Thus, QSH insulators are good starting materials to achieve QAHE. Several studies have predicted that thin films of groups IV (Sn) [11–13] and V (Bi, Sb) [6, 14–17] support QSH phases which can also be achieved via chemical functionalization [17, 18]. Besides group IV and V elements, it was also predicted that [19–21] III-V honeycombs support the QSH phase in both freestanding and functionalized cases. These results paved the way for finding QAHE phases. Studies have shown that QAHE were found to exist in functionalized group IV [22] and V [17, 18, 22] thin films. In addition, first-principles calculations show QAHE in fluorinated [23] and chemically functionalized [24] III-V honeycombs. Moreover, several theoretical studies have predicted that transition metals doping in honeycombs can induce QAH phases [17, 25–27]. This has been experimentally realized via Cr and V doping [8, 28, 29]. Supported by the finding that III-V honeycomb materials are QSH insulators [19] and the theoretical prediction that doping a magnetic material can induce magnetism [10], we replace the group III element with a transition metal (M=Ti, Zr, and Hf). Transition-metal carbides MC (M=Zr and Hf) [30] and transition-metal halides MX (M=Zr and Hf) [31] are also another family of materials predicted to exist as QSH insulators. However, its potential to support QAHE has not been explored yet. Motivated by these findings, we predict the electronic properties of transition-metal pnictides MPn (M=Ti, Zr, and Hf; Pn=Sb and Bi) to exhibit the QAH phase. In this work, we employ first-principles calculations to predict the ability of transition metals (M =Ti, Zr, and Hf) to induce intrinsic magnetism on Bi/Sb honeycombs. We examine both buckled and planar cases and identify the phase changes due to strain. The QAH phases are verified by calculating the Chern number and observing band inversion.

## Results and Discussions

**Fig. 1:**
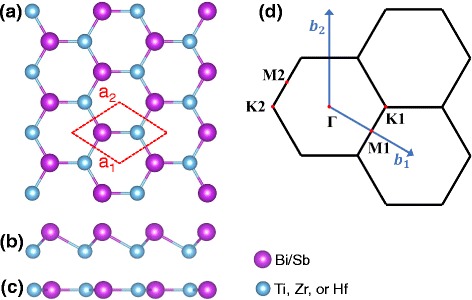
**a** Crystal structure of M-Sb/Bi honeycomb. **b**, **c** Side views of buckled and planar structures, respectively. **d** The first Brillouin zone (BZ) with high-symmetry points

Similar to pure Bi honeycomb (with two atoms in the unit cell) which can adopt both buckled and planar structures, our material is obtained by replacing half of Bi by a transition metal [e.g., Ti, Zr, and Hf] in the unit cell. The top view of M-Bi/Sb with an outlined 1 ×1 unit cell is shown in Fig. 1a, while the side views of buckled and planar M-Bi/Sb honeycombs are shown in Fig. 1b, c, respectively. The corresponding first Brillouin zone (BZ) labeled with high-symmetry points is shown in Fig. 1d.

**Fig. 2:**
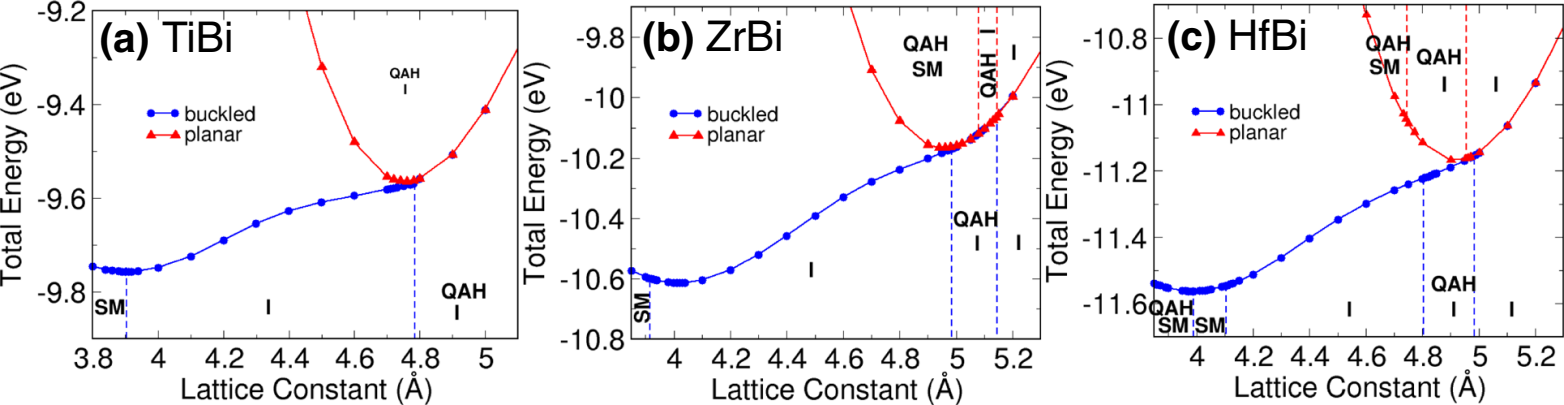
Phase diagram of **a** TiBi, **b** ZrBi, and **c** HfBi showing the total energy at different lattice constants. The diagram is divided into various regions labeled as QAH (quantum anomalous Hall phase), I (insulator), and SM (semi-metal). Blue circles and red triangles represent buckled and planar cases, respectively

We study the stability of honeycombs and the effect of strain by varying the lattice constant and allowing the atoms to relax for both buckled and planar cases. Next, we identified their topological phases under different strains through the Chern number calculations. The result is illustrated via a phase diagram as presented in Fig. 2. The energy curves for TiBi, ZrBi, and HfBi are shown in Fig. 2a-c, respectively. We found that MBi honeycombs possess the low-buckled and planar phases. Through these figures, we identify the equilibrium lattice constants for further analysis. The figure also shows that buckled MBi is the energetically favored structure. However, most of the QAH phases are observed when the strain is increased which transforms the material from buckled to planar honeycombs. It should also be noted that QAH phases could be observed in buckled HfBi but only within a small range of lattice constants [see Fig. 2c].

**Fig. 3:**
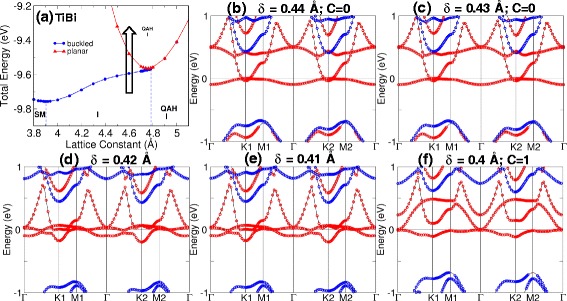
Phase transition after varying the buckled distance. **a** Phase diagram of TiBi at *a*=4.6 Å. The arrow shows the path of the transition. **b**–**f** The band structure transition as the buckling distance (*δ*) was reduced from 0.44 to 0.4 Å. The transition occurs at *δ*=0.41 Å

**Fig. 4:**
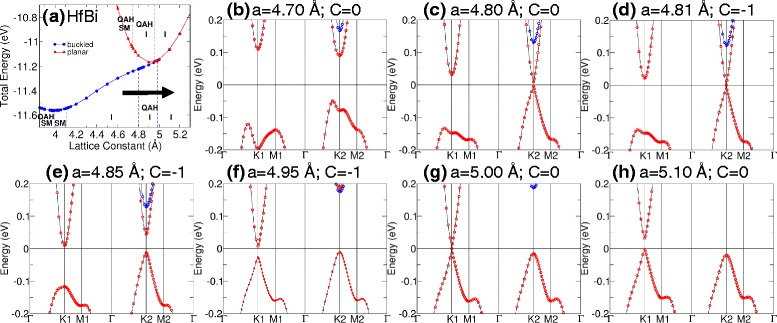
Phase transition after varying the lattice constant. **a** Phase diagram of buckled HfBi. The arrow shows the path of the transition. **b**–**h** The band structure transition as the lattice constant was increased from 4.7 to 5.1 Å

Tables 1 and 2 show the equilibrium lattice constants for M-Bi and M-Sb structures. The associated band gap, magnetic moment, phase, and material classification are also indicated. QAHE is present when the calculated Chern number, C, is a non-zero integer. The band gap is calculated as the difference between the lowest unoccupied and the highest occupied bands. Our calculations show that the QAH insulator phase can be found in planar TiBi and HfBi with band gaps of 15 and 7 meV, respectively. Moreover, phase transition can be induced in TiBi by varying the buckled distance [see Fig. 3] and by inducing strain in buckled HfBi [Fig. 4]. In TiBi, we find that the band crossings due to the varying of buckling distance occur at low symmetry points shown in Fig. 3d; while in HfBi, we observed the two band crossings (critical transition points) first at K2 (*a*=4.8 Å) and then at K1 (*a*=5.0 Å) due to strain in Fig. 4c, g.

**Table 1:** Calculated equilibrium lattice constants, system band gaps, magnetic moment, and topological phase of planar and buckled M-Bi honeycombs

	M-Bi	Lattice constant (Å)	Band gap (meV)	Phase	Classification	Mag. (*μ*_*B*_)
Planar	TiBi	4.76	15	QAH	Insulator	1.050
	ZrBi	4.96	−3	QAH	Semi-metal	1.005
	HfBi	4.92	7	QAH	Insulator	0.947
Buckled	TiBi	3.9	−12	–	Semi-metal	1.085
	ZrBi	4.01	10	–	Insulator	1.046
	HfBi	3.98	−54	QAH	Semi-metal	1.005

**Table 2:** Calculated equilibrium lattice constants, system band gaps, magnetic moment, and topological phase of planar and buckled M-Sb honeycombs

	M-Sb	Lattice constant (Å)	Band gap (meV)	Phase	Classification	Mag. (*μ*_*B*_)
Planar	TiSb	4.64	−70	QAH	Semi-metal	1.004
	ZrSb	4.84	8	–	Insulator	0.996
	HfSb	4.82	−50	–	Metal	0.948
Buckled	TiSb	3.81	256	–	Insulator	1.007
	ZrSb	3.94	230	–	Insulator	1.003
	HfSb	3.92	−59	QAH	Semi-metal	0.979

**Fig. 5:**
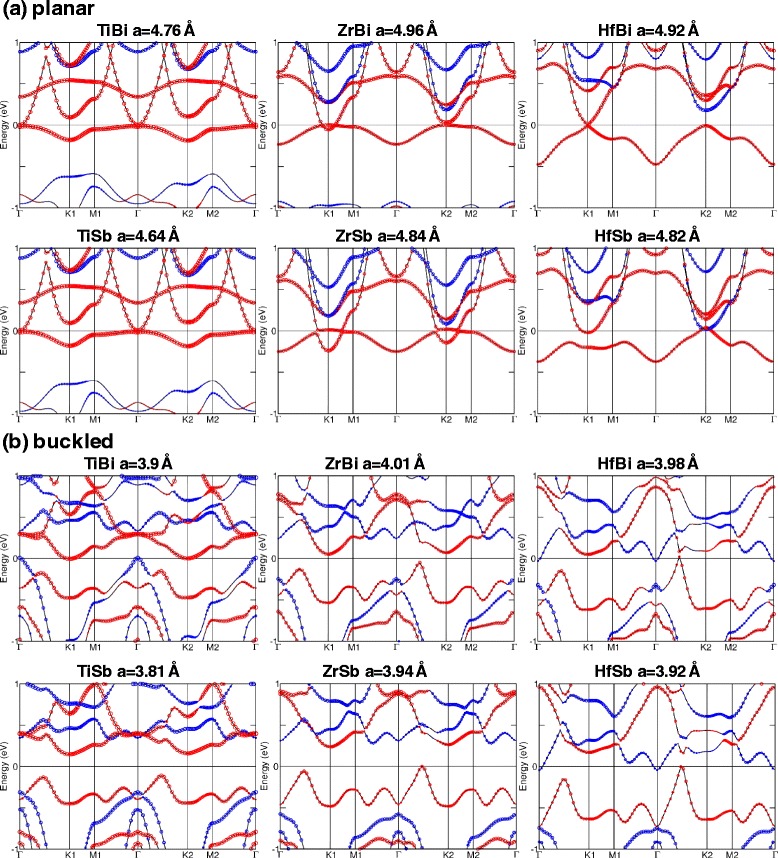
Electronic band structures of M-Pn (M=Ti, Zr, and Hf; Pn=Sb and Bi) at their equilibrium lattice constants for **a** planar and **b** buckled cases. The equilibrium lattice constants are given above the band structure. Red and blue circles indicate +*s*_*z*_ and −*s*_*z*_ contributions, respectively

Figure 5a, b shows the electronic band structures at equilibrium lattice constants for M-Bi and M-Sb in planar and buckled structures, respectively. The red and blue circles are the spin up and spin down contributions, respectively. The QAH phase (with *C*=1) with largest band gap is 15 meV observed in planar TiBi. Planar HfBi is also a QAH insulator with a small band gap of 7 meV (with *C*=−1). However, in a buckled form, HfBi is a semi-metal with a high *C*=−3. On the other hand, buckled ZrBi, TiSb, ZrSb, and planar ZrSb are found to be trivial insulators.

**Fig. 6:**
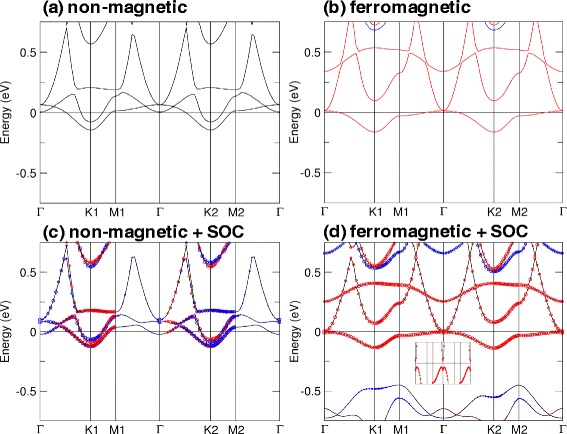
Electronic band structures of planar TiBi film at *a* = 4.76 Å for non-magnetic calculations (**a**) without SOC and (**c**) with SOC as well as ferromagnetic calculations (**b**) without SOC and (**d**) with SOC. Red and blue circles indicate +*s*_*z*_ and −*s*_*z*_ contributions, respectively, for (**c**) non-magnetic (**d** ferromagnetic) calculations with SOC

The nature of QAHE can be further understood by examining the effects of SOC in non-magnetic and ferromagnetic calculations. For this purpose, we chose planar TiBi (with *a*=4.76 Å) as the exemplar. The band structures obtained in non-magnetic and ferromagnetic calculations with and without SOC are shown in Fig. 6. Our calculations show that this structure has a magnetic moment of 1.05 *μ*_*B*_ per unit cell which is mainly contributed by Ti atoms. In the non-magnetic calculations, we find that the system is metallic [Fig. 6a, c]. We can observe in Fig. 6b that a net magnetic moment can be induced due to ferromagnetic ordering which is influenced by the transition metal, Ti. Furthermore, the system now has gapless spin-up states (red lines) and gapped spin-down states, and by applying SOC to the ferromagnetic calculation, a gap of 15 meV is then obtained. This shows that the band inversion is induced by SOC and the gap opening results in QAHE.

**Fig. 7:**
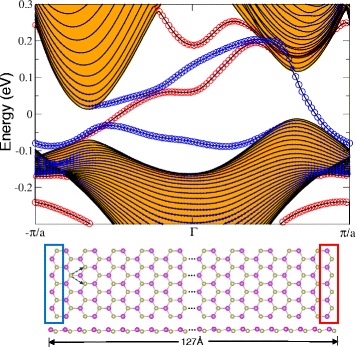
Band structure along the edge of buckled HfBi zigzag nanoribbon with *a*=4.9 Å and the width of 127 Å. Blue (red) circles indicate the contribution from the left (right) edges. The bulk bands are denoted by the orange-filled region

Finally, we inspect the edge band spectrum of planar HfBi honeycomb for the presence of edge states using tight-binding Hamiltonians derived via Wannier functions. We constructed HfBi ribbons with zigzag edges and width of 127 Å as shown in Fig. 7. The figure also confirms the presence of edge states denoted by and proportional to the size of the red and blue circles which represent the right and left edges, respectively. The separate edge states are due to the asymmetry of the right and left zigzag edges.We can also observe an odd number of edge band crossing the fermi level. We find that this number is the same as the absolute value of the Chern number, further confirming the QAH phase in planar HfBi.

We have further calculated the phonon spectrum for each system and noted that these systems possessed negative frequency. Thus, the aforementioned systems would need a substrate to stabilize. We also noted that the aforementioned calculations were done using a one by one unit cell, and the materials with ferromagnetic (FM) configuration is the most stable state. However, for a larger supercell, we found that FM still has a lower energy than the anti-ferromagnetic (AFM) configuration in the buckled cases, while both FM and AFM configurations are degenerate in energy in the planar cases.

## Conclusions

To summarize, our first-principles calculations predict that the replacement of transition metals (Ti, Zr, and Hf) on Sb or Bi honeycomb films could potentially exhibit the QAH phase. Although these materials are energetically more stable in their buckled form, transforming it to planar form yields the QAH phase in a quite reasonable range of lattice constants. Such phase can also be induced by varying the buckling distance and by applying strain as should in our calculated phase diagrams. We find that planar TiBi and HfBi structures exist as QAH insulators with a band gap of 15 and 7 meV, respectively. These findings offer another way of realizing the QAH phase in honeycomb materials which could potentially be of use in spintronic applications.

## Methods/Experimental

First-principles calculations within the density functional theory (DFT) framework were performed using the generalized gradient approximation (GGA) [32–36] and projector-augmented-wave (PAW) [37] method as implemented in the Vienna Ab-Initio Simulation Package Version 5.3 (VASP) [38, 39]. The kinetic energy cutoff was set to 350 eV and the crystal structures were optimized until the residual forces were no greater than 5×10^−3^ eV/Å. The self-consistency criteria for convergence was set at 10^−6^ eV for electronic structure calculations with or without spin-orbit coupling. We simulate a thin film by inserting a vacuum layer of at least 20 Å along the *z* direction on a sampled 2D Brillouin zone of 24×24×1 Gamma-centered Monkhorst-Pack grids [40]. We calculated the maximally localized Wannier functions using the WANNIER90 package [41] which were then used to calculate edge states. The topological phases were identified by calculating the Chern number using Z2Pack package [42, 43] which utilizes a technique that will track hybrid Wannier charge centers.
